# Effect of Collateral Flow on Catheter-Based Assessment of Cardiac Microvascular Obstruction

**DOI:** 10.1007/s10439-022-02985-2

**Published:** 2022-05-31

**Authors:** Mirunalini Thirugnanasambandam, Sabrina Frey, Yannick Rösch, Alberto Mantegazza, Francesco Clavica, Robert S. Schwartz, Nikola Cesarovic, Dominik Obrist

**Affiliations:** 1 ARTORG Center for Biomedical Engineering Research, University of Bern, Freiburgstrasse 3, 3010 Bern, Switzerland; 2 CorFlow Therapeutics AG, Baar, Switzerland; 3 Department of Biomedical Engineering, Pennsylvania State University, University Park, PA USA; 4 Department of Health Science and Technology, ETH Zurich, Zurich, Switzerland; 5 Cardiosurgical Research Group, Department of Cardiothoracic and Vascular Surgery, German Heart Center Berlin, Berlin, Germany

**Keywords:** Coronary circulation, Controlled flow infusion, MVO, STEMI, Balloon catheter

## Abstract

Cardiac microvascular obstruction (MVO) associated with acute myocardial infarction (heart attack) is characterized by partial or complete elimination of perfusion in the myocardial microcirculation. A new catheter-based method (CoFI, Controlled Flow Infusion) has recently been developed to diagnose MVO in the catheterization laboratory during acute therapy of the heart attack. A porcine MVO model demonstrates that CoFI can accurately identify the increased hydraulic resistance of the affected microvascular bed. A benchtop microcirculation model was developed and tuned to reproduce *in vivo* MVO characteristics. The tuned benchtop model was then used to systematically study the effect of different levels of collateral flow. These experiments showed that measurements obtained in the catheter-based method were adversely affected such that collateral flow may be misinterpreted as MVO. Based on further analysis of the measured data, concepts to mitigate the adverse effects were formulated which allow discrimination between collateral flow and MVO.

**Supplementary Information:**

The online version contains supplementary material available at 10.1007/s10439-022-02985-2.

## Introduction

Epicardial flow is obstructed during acute myocardial infarction due to local coronary artery thrombosis (primary lesion). The myocardium downstream from this epicardial lesion is not perfused unless collateral circulation is present, functioning to bypass the occluded vessel. Epicardial perfusion is re-established following successful coronary intervention, typically with stent placement. However, despite reperfusion with good epicardial flow, it has been found that 40–60% of ST-segment Elevation Myocardial Infarction (STEMI) patients have occlusion of the microvasculature,^7,9^ known as microvascular obstruction (MVO).^1^ MVO, when present, has marked impact on both early and late prognosis.^4^ The diagnosis of MVO is the target of this study.

Late Gadolinium Enhanced Cardiac Magnetic Resonance Imaging (LGE-CMRI)^3^ is the gold-standard technique for MVO quantification. However, MRI is not practical in the acute setting, and therefore, methods which allow for both qualitative and quantitative MVO diagnosis in the catheter lab immediately following treatment of the primary lesion would be very useful. Recently, a catheter-based method has been developed and tested to quantify resistance in the myocardial microvascular bed distal to the primary lesion.^16^ It is applied after recanalization of the epicardial vessel. This method (CoFI, controlled flow infusion) uses a multi-lumen balloon catheter (occlusion–infusion catheter) connected to an infusion pump, and a pressure guidewire to measure pressure distal to the balloon. Temporary balloon occlusion at the site of the recanalized primary lesion, is followed by infusion of an isotonic buffered solution distal to the balloon at a series of controlled flow rates. Distal coronary pressure measurements during this infusion provide quantitative information on coronary resistance, which can be estimated from the coronary artery pressure at known flow rates. This occlusion–infusion sequence (OIS) is able to detect increased microvascular resistance from MVO in pig models.^15,16^

However, a strong quantitative correlation between measured resistance and amount of MVO still needs to be established, because vascular resistance measurement with OIS may be affected by collateral flow bypassing the primary lesion. In humans, collateral vessels in the coronary circulation are common.^18^ The structure and number of collaterals varies among individuals and is typically not known a priori in patients. Therefore, it is important to understand their characteristic behavior as an alternative source of blood supply to ischemic myocardial tissue^18,20^ and to be able to distinguish effects of collaterals from other pathologies (e.g. MVO).

The objectives of the present study were to develop a benchtop model of the coronary circulation and to reproduce the results obtained in the pig model (assuming no collateral flow). This model would then be used to systematically study the effect of collateral flow. In addition, the model was used to assess the effect of mixing of water-like infused fluid with the blood in the vascular bed. This mixing during OIS decreases the fluid viscosity and thereby affects the hydraulic resistance.

Only few benchtop models of the coronary circulation have been described so far. Early models^10,17^ used aortic pressure to drive coronary flow, but did not account for the effect of ventricular contraction on vascular resistance. This limitation was mitigated in later models,^5,12^ where the dynamics of coronary flow was controlled by an intramyocardial pump element. The coupling of systemic and coronary lumped parameter models in Geven *et al*.^5^ reproduced physiological coronary waveforms. The present model for coronary circulation extends previous models and features elements for modelling collateral flow and MVO and allows access for the occlusion–infusion catheter used in OIS.

## Methods

### Study Methodology

To meet the objectives of this study, the following methodology was adopted: First, a multi-scale benchtop model was designed by combining a validated left-heart mock loop with a novel coronary model comprising a series of adjustable impedance elements to model the hemodynamics of the coronary vascular tree. Second, this benchtop model was validated by demonstrating that it could reproduce physiological waveforms for coronary flow and that it was possible to find settings for the impedance elements of the coronary model such that OIS data obtained from pig model (with and without MVO) could be reproduced. Third, the validated model was used to extrapolate the baseline pig data for systematically increased collateral flow rates. To distinguish between effects for collateral flow and fluid mixing, these experiments were first carried out only with water (to exclude effects of fluid mixing) and then repeated with a blood mimicking fluid and water to include mixing effects.

### Occlusion–Infusion Sequence (OIS)

**Figure 1:**
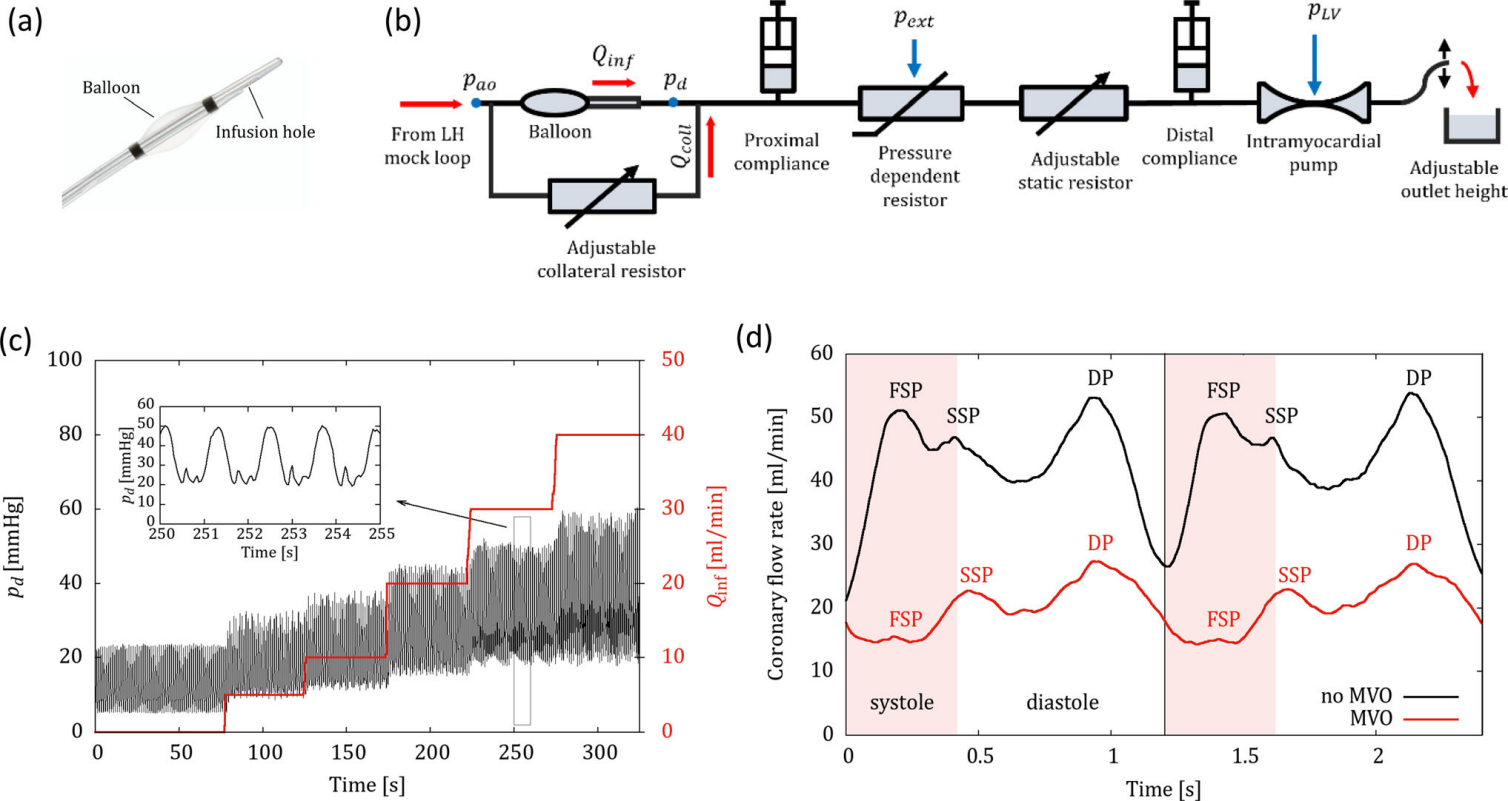
(a) Tip of the occlusion–infusion catheter used for the occlusion–infusion Sequence (OIS). (b) Schematic of the coronary model with impedance elements (${p}_{ao}$: aortic pressure, ${p}_{LV}$: Left ventriclular pressure, ${p}_{d}$: distal pressure, ${p}_{\text{ext}}$: Hydrostatic pressure applied to increase PDR resistance, ${Q}_{\text{inf}}$: infusion flow rate, ${Q}_{\text{coll}}$: collateral flow rate). (c) Typical flow-pressure response generated by the OIS (black: distal pressure ${p}_{d}$ , red: influsion flow rate ${Q}_{\text{inf}}$). The inset shows the shape of the pressure waveform for four heartbeats at approximately 50 bpm. (d) Coronary flow rate measured at the LAD phantom without balloon inflation and without collateral flow (black: no-MVO; red: MVO). (*DP* diastolic peak; *FSP* first systolic peak; *SSP* second systolic peak; red bar: systolic phase).

This study is based on the OIS, a catheter-based method to determine flow and pressure at the location of the (previously recanalized) primary lesion. To this end, a custom rapid-exchange, occlusion–infusion catheter (CorFlow Controlled Flow Infusion™ Rapid Exchange Catheter, CoFI, CorFlow Therapeutics, Baar, Switzerland) is advanced over a pressure sensing guidewire (OptoWire Deux, OptoSens, Canada). The catheter features an inflatable balloon which is placed at the primary lesion site (Fig. 1a). When inflated, the balloon blocks blood flow to the distal vasculature. Distal to the blocking balloon, isotonic buffered fluid is infused through an end-hole lumen of the catheter at well-defined flow rates of ${Q}_{\text{inf}}$ = 5, 10, 20, 30, and 40 mL/min for 30 s each using a peristaltic pump (LiveTec LiveCool Infusion Pump, livetec Ingenieurbüro GmbH, Lörrach, Germany). This infusion is assumed to be at stepwise steady flow rates, although the peristaltic pump design (roller pump) introduces minor oscillations in the infusion flow rate. There is sufficient time scale separation between these oscillations and the OIS steps, such that they are not expected to affect the results. Simultaneous with the infusion, the pressure ${p}_{d}$ distal to the balloon is measured with the pressure sensing guidewire at a rate of 25Hz.

The OIS yields a flow-pressure response curve (Fig. 1c) which is used to characterize the coronary circulation. The curve Fig. 1c in was obtained without any collateral flow. Therefore, the pulsatile waveform of the pressure signal was only due to to the myocardial contractions introduced through the IP element. The ratio of pressure ${p}_{d}$ and flow rate ${Q}_{\text{inf}}$ can be interpreted as a measure for the hydraulic resistance of the vascular bed downstream of the balloon. Accordingly, the measured pressure increases stepwise with increasing flow rates. The non-zero pressure at zero pump flow is known as the coronary wedge pressure.^18,19^

The pulsatile distal pressure ${p}_{d}$ is decomposed into its tonic (steady) and phasic (oscillating) components. We quantify these components by the temporal mean (tonic pressure, ${p}_{\text{tonic}}$), the standard deviation (phasic pressure, ${p}_{\text{phasic}}$) and the oscillation amplitude ${p}_{osc}$ which are defined as

$$ {\text{Tonic}}\,{\text{pressure}}:p_{{{\text{tonic}}}} = \frac{1}{N}\mathop \sum \limits_{n} p_{d,n} $$

$$ {\text{Phasic}}\,{\text{pressure}}:\,p_{{{\text{phasic}}}} = \sqrt {\frac{1}{N - 1}\mathop \sum \limits_{n} \left( {p_{d,n} - p_{{{\text{tonic}}}} } \right)^{2} } $$

$$ {\text{Oscillation}}\,{\text{amplitude}}:\,p_{{{\text{osc}}}} \, = \,\max \left( {p_{d,n} } \right) - \min \left( {p_{d,n} } \right) _{ }^{ } $$

where ${p}_{d,n}$ is the *n*th element in a vector of length *N* which contains all measured values of ${p}_{d}$ at a specific infusion flow rate ${Q}_{\text{inf}}$. The interval of interest for the vector ${p}_{d}$ was adjusted to exclude transient effects due to the stepwise increase in flow rate. The oscillation amplitude ${p}_{\text{osc}}$ is only used for model tuning, because this quantity is easier to monitor during the experiment than the phasic pressure.

### Animal Model

Animal housing and experimental protocols were approved by the Cantonal Veterinary Office, Zurich, Switzerland, under License ZH 219/16, and were performed in accordance with Swiss animal protection law and ordinance. Animal housing and experimental procedures also conformed to the European Directive 2010/63/EU of the European Parliament and the Council of 22 September 2010 on the Protection of Animals Used for Scientific Purposes and to the Guide for the Care and Use of Laboratory Animals.

Coronary hemodynamic response without and with MVO were evaluated sequentially with OIS in a pig model. Data used to tune the benchtop model was based on experimental results of one animal, as a part of a larger study to investigate development and progression of MVO in a porcine model of STEMI.

In brief, a domestic pig (60 kg) received premedication with ketamine (20 mg/kg), azaperone (1.5 mg/kg) and atropine (0.75 mg) intramuscularly. After loss of postural reflexes, the anesthesia was deepened by a bolus injection of propofol (1–2 mg/kg) to facilitate intubation. Anesthesia was maintained with 2–3% isoflurane and propofol (2–5 mg/kg/h) for the remainder of the experiment. Amiodarone (2–3 mg/kg bolus IV) was administered to stabilize the heart rhythm. Pain management included fentanyl infusion (0.02 mg/kg/h) for the duration of the procedure. Following stent placement, the OIS catheter and pressure guidewire were introduced into the left anterior descending artery (LAD) via femoral access. OIS was carried out to obtain a physiological baseline flow-pressure response. To obtain OIS data in presence of MVO, the LAD was then occluded for 90 minutes to achieve myocardial infarction as described in Ref ^6^. Following the occlusion, reperfusion was initiated as the balloon was removed. MVO then resulted in the distal microvascular territory without infusion of any micro-emboli. OIS was repeated and the corresponding flow-pressure response was used as baseline data for MVO. The presence of MVO was then confirmed by gold standard quantification with LGE-CMRI^3^ which was performed within 6 hours after reperfusion.

For the animal used in the present study, we identified the MVO volume as 2.5% of the global LV wall. We assume here that these OIS measurements were not affected by collateral flow, because pigs are known to have no (or only very few) collateral vessels.^14^

### Benchtop Model

A multi-scale benchtop model was developed to systematically assess the hemodynamics in the coronary circulation with and without MVO and collateral flow. The model consisted of two sub-circuits: (a) The left-heart mock loop, which provided appropriate inflow conditions at the coronary ostia and the left-ventricular pressure used to model compression of the intramyocardial vessels; (b) the coronary model, which was connected to the left-heart mock loop through an ostium.

#### Left-Heart Mock Loop

The left-heart mock loop^8^ comprised a computer-controlled piston pump and a PMMA chamber representing the left ventricle (LV) which was connected to a silicone aortic root phantom with a mechanical heart valve. The anatomically accurate aortic root phantom included three sinus portions with two coronary ostia (one ostium was connected to the coronary flow loop; the second ostium was blocked). The aortic root was connected distally to a compliance chamber and an adjustable resistance element to model arterial compliance and systemic resistance, respectively. The flow returned to a PMMA model of the left atrium which was connected to the LV model by a second mechanical heart valve.

Pressures in the mock loop were measured with PBMN pressure transmitters (Baumer Electronics, Frauenfeld, Switzerland) and cardiac output was monitored with transit-time ultrasonic flow probes (Transonic Clamp-on Flow Sensor 16PXL, Transonic Systems Inc., Ithaca, NY, USA).

The pump, the compliance and the resistance elements of the left-heart mock loop were tuned to a heart rate (HR) of 50 bpm and aortic pressures (BP) of 55/80 mmHg, which closely matched the values for the healthy pig (HR = 50bpm, BP = 52/82 mmHg). This configuration was maintained also for experiments with MVO to reduce the number of varied parameters, although the animal with MVO had a higher heart rate (HR = 67 bpm, BP = 53/78 mmHg).

#### Coronary Model

The coronary model comprised several impedance elements to model the complex dynamics of the coronary circulation (Fig. 1b).

Proximally, the coronary model was connected to a coronary ostium of the left-heart model which imposed the aortic pressure ${p}_{\text{ao}}$ at the upstream end of the model. The pulsatile nature of the aortic pressure induced a pulsatile flow in the coronary model. The tubing connected to the coronary ostium modelled the first generations of the left coronary arterial tree including the LAD which was assumed to be the location of the (recanalized) primary lesion. This location was accessible with the OIS catheter through a separate port.

The LAD model was bypassed by a second tube including an adjustable resistive element. This bypass modelled a collateral branch which could provide a collateral flow rate ${Q}_{\text{coll}}$ to the coronary model even when the balloon of the OIS catheter occluded the flow in the main branch. The hydraulic resistance in the collateral vessel branch could be manually adjusted by a clamp, which was placed around the tubing. The collateral resistor was fully closed to model the situation in the pig (${Q}_{\text{coll}}$ = 0 mL/min) and it was gradually opened to create different levels of collateral flow.

The distal vascular tree including the associated microvasculature (with or without MVO) was modeled with the following tunable impedance elements (from left to right in Fig. 1b):

- *Proximal compliance element* to model the vascular compliance of the large epicardial coronary arteries composed of a syringe filled with fluid and air that was connected to the main flow path (Supplementary Fig. S1c). The compliance could be adapted by changing the air volume in the syringe.
- The *pressure dependent resistor (PDR)* element created a hydraulic resistance which changed with the coronary perfusion pressure (Supplementary Fig. S1a,b). It comprised two channels that were separated by a 20 µm PDMS membrane. The fluid in the coronary phantom flowed through the lower channel, while the upper control channel was connected to a water column imposing a hydrostatic pressure ${p}_{ext}$. By choosing ${p}_{ext}$ appropriately, the PDMS membrane was deflected downwards at low perfusion pressures, resulting in a reduction of the lower channel cross-section and an increase in hydraulic resistance, whereas the lower channel opened at higher perfusion pressures decreasing the resistance.
- An *adjustable static resistor* in the form of a clamp was used to model the hydraulic resistance of the coronary tree (Supplementary Fig. S1e). This resistor was also used to reflect the increase in hydraulic resistance due to MVO.
- The *distal compliance element* was built like the proximal compliance (Supplementary Fig. S1c). It was used to model the compliance of the intramyocardial vessels and the microcirculation.
- The *intramyocardial pump (IP)* element modeled the effect of ventricular contraction on the hydraulic resistance of the endocardial vessels (Supplementary Figure S1d). Its design was motivated by the adaptive element used in Geven *et al*.^5^ It comprised a thin-walled collapsible tube (Penrose drain tube^2^) which was placed in a fluid-filled chamber. This chamber was connected to the LV such that the chamber pressure corresponded to the LV pressure ${p}_{LV}$ (which was used as an approximation to the intramyocardial pressure). High ${p}_{LV}$ during systole compressed the thin-walled tube and increased its hydraulic resistance, while the tube expanded during diastole lowering the resistance.
- The distal end of the coronary model was open such that the fluid drained into a bucket at atmospheric pressure. This drain can be interpreted as a model for the coronary sinus. By elevating or lowering the open end a hydrostatic pressure could be imposed.

A transit-time ultrasonic flow probe (Transonic Clamp-on Flow Sensor 3PXL, Transonic Systems Inc., Ithaca, NY, USA) was placed at the inflow of the coronary model to measure the coronary flow rate. To assess the amount of collateral flow during OIS, the flow probe was placed around the collateral branch.

#### Working Fluids

Water was used in all experiments as the fluid infused through the OIS catheter. Its dynamic viscosity of 0.001 Pa s corresponded to that of the isotonic buffered solutions that were used in the pig experiments.

In a first step, water was also used in the left-heart mock loop and in the coronary model to exclude effects of fluid mixing. Then, blood mimicking fluid (BMF) was used in the left-heart mock loop and coronary model to study the effect of viscosity changes in the coronary circulation. BMF was prepared at room temperature as a 40%/60% (by weight) glycerin/water mixture to match blood viscosity (0.0035 Pa s).

### Experimental Protocol

Each experiment with the described model consisted of four steps:

1. The benchtop model was tuned with closed collateral branch (${Q}_{\text{coll}}$= 0 mL/min) such that the OIS response of the model matched the OIS response of the healthy pig. This tuning was done by manually adjusting the impedance elements following the algorithm described in the Online Appendix.
2. The OIS was repeated for different collateral flow rates by adjusting the resistance element at the collateral vessel (while the settings of the other impedance elements were held constant).
3. The model tuning was adapted to data from the (same) animal with MVO and ${\mathrm{Q}}_{\mathrm{coll}}$= 0 mL/min.
4. The OIS was repeated for ${\mathrm{Q}}_{\mathrm{coll}}>0$ mL/min (like in step 2).

In the present study, this experimental protocol was carried for data from one animal. The experiments were done first with water, and second, with BMF.

## Results

### Coronary Waveform

The coronary flow rate was measured in the LAD phantom after the model (with BMF) had been tuned to the healthy animal (Fig. 1d). The coronary waveform in the model exhibited pulsatile behavior typical for coronary flow in the LAD, with maximum flow during diastole and minimum flow during systole.^13^ The mean flow rate was 45 mL/min. After tuning the model to the MVO animal data, the mean coronary flow rate reduced to 20 mL/min.

### OIS with Water

**Figure 2:**
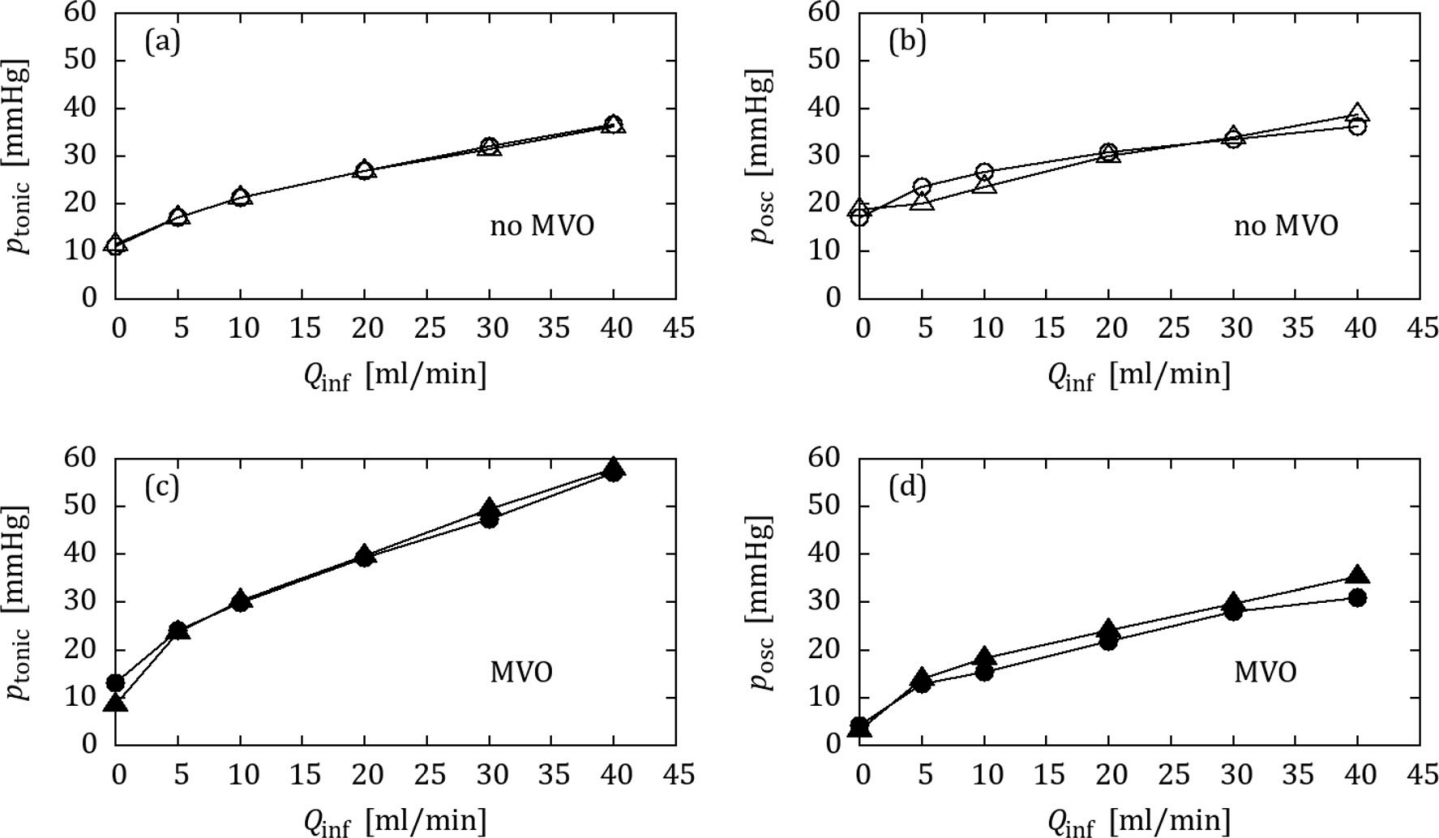
OIS data from the animal trial (△,▲) and from the benchtop model (○,●) with water: (a, b) tonic pressure and oscillation amplitude without MVO (open symbols), (c, d) tonic pressure and oscillation amplitude with MVO (filled symbols).

Figure 2 shows tonic pressures and oscillation amplitudes after tuning the benchtop model to OIS data from the healthy pig (Figs. 2a, 2b) and the pig with MVO (Figs. 2c, 2d). The collateral bypass was blocked in the benchtop model (${Q}_{\text{coll}}$ = 0 mL/min). The good match between animal and benchtop data indicates that it was possible to find a setting for the impedance elements for which the benchtop reproduced the *in vivo* OIS response without MVO and a second setting with increased peripheral impedance which reproduced the OIS response with MVO.

**Figure 3:**
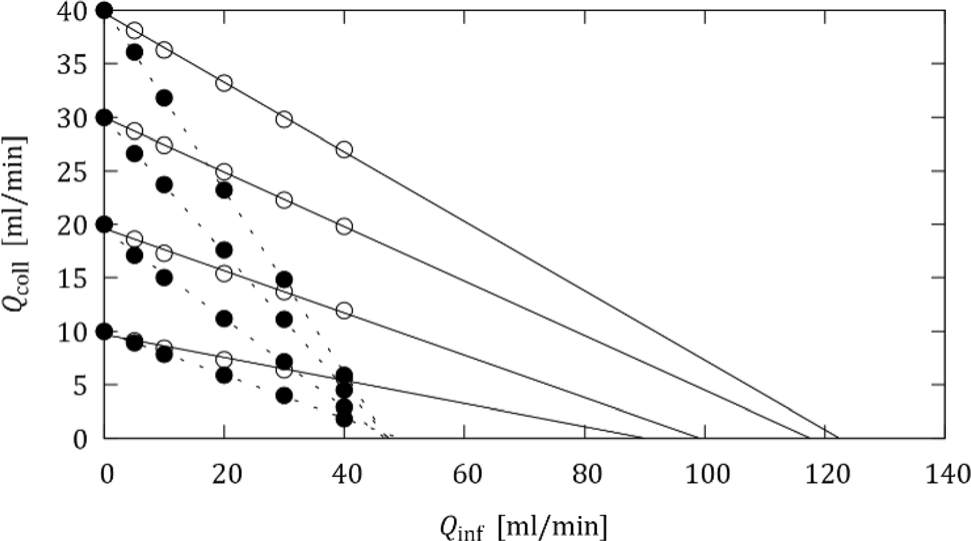
Collateral flow rate ${Q}_{\text{coll}}$ in function of ${Q}_{\text{inf}}$. Experiments in no-MVO configuration (open symbols; solid line) and with MVO (filled symbols; dashed lines) for different initial collateral flow rates (${Q}_{\mathrm{coll},0}$= 10, 20, 30, 40 mL/min).

After this tuning, the OIS was repeated for different initial collateral flow rates ${Q}_{\text{coll,0}}$ = 10, 20, 30 and 40 mL/min, which were set by adjusting the collateral resistor while there was no infusion flow through the OIS catheter (${Q}_{\text{inf}}$ = 0 mL/min) and the balloon occluded the LAD model. The respective setting of the collateral resistor was held constant for the subsequent OIS. Figure 3 shows that the mean collateral flow rate ${Q}_{\text{coll}}$ decreased during the OIS, because an increase in ${Q}_{\text{inf}}$ also increased the distal pressure ${p}_{d}$ which reduced the pressure difference across the collateral branch and led to a reduction in ${Q}_{\text{coll}}$.

The nearly linear decay of ${Q}_{\text{coll}}$ in Fig. 3 suggested a linear extrapolation of the measured data points beyond the maximum flow rate of ${Q}_{\text{inf}}$ = 40 mL/min (which was chosen for safety reasons, in order not to create excessive pressures in the coronary tree). These extrapolations suggest that collateral flow could be suppressed if ${Q}_{\text{inf}}$ was increased to 90 to 120 mL/min for the healthy configuration and to 45 to 50 mL/min for the MVO configuration.

**Figure 4:**
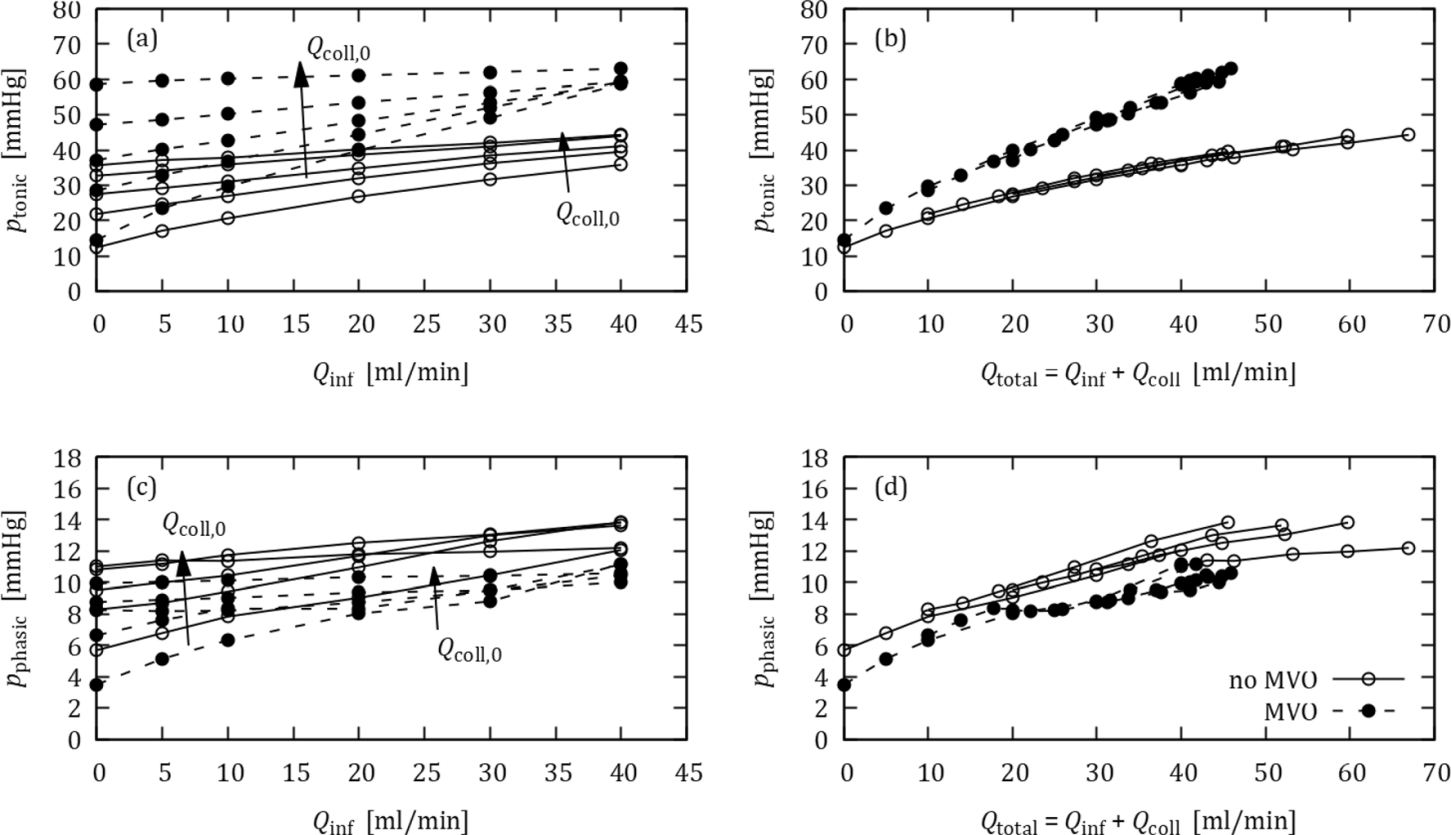
OIS responses for different initial collateral flow rates ${Q}_{\mathrm{coll},0}$ (0, 10, 20, 30, 40 mL/min) with (dashed lines) and without MVO (solid lines): (a) tonic pressure against ${Q}_{\text{inf}}$, (b) tonic pressure against ${Q}_{\mathrm{total}}$ = ${Q}_{\mathrm{inf}}$ + ${Q}_{\mathrm{coll}}$, (c) phasic pressure against ${Q}_{\text{inf}}$, (d) phasic pressure against ${Q}_{\mathrm{total}}$.

Figure 4 summarizes the OIS responses for different initial collateral flow rates ${\mathrm{Q}}_{\mathrm{coll},0}$. In general, higher collateral flow and/or the presence of MVO led to higher tonic pressures (Fig. 4a). In clinical practice, it is therefore difficult to distinguish between effects of collateral flow and MVO based on tonic pressure data from OIS.

Figure 4b shows the same data as in Fig. 4a with a modified abscissa: The tonic pressure is now plotted against the total distal flow rate ${Q}_{\text{total}}$ = ${Q}_{\text{inf}}$ + ${Q}_{\text{coll}}$ where ${Q}_{\text{coll}}$ was taken from Fig. 3 (note that ${Q}_{\text{coll}}$ is typically not known in *in vivo* measurements). This modification results in a collapse of the curves for different initial collateral flow ${Q}_{\text{coll,0}}$ and separates the curves for MVO and no-MVO. The increased slope of the MVO curves illustrates the increased hydraulic resistance of the distal vascular bed with MVO.

Figure 4c illustrates the effect of collaterals on the phasic pressure. Like the tonic pressure, the phasic pressure increased with increasing ${Q}_{\text{coll,0}}$. However, presence of MVO reduced the phasic pressure, which is opposite to the behavior for the tonic pressure. Figure 4d shows the phasic pressure against ${Q}_{\text{total}}$ = ${Q}_{\text{inf}}$ + ${Q}_{\text{coll}}$ resulting in a separation of MVO from no-MVO data.

### OIS with BMF

The previous results were obtained with water alone and neglected effects of changing viscosity due to mixing of infused water with blood. Therefore, the experiments were repeated with BMF to include mixing effects. However, for the initial tuning of the model to the animal data, only the left-heart mock loop was filled with BMF, whereas the coronary model was filled with water. The two fluids did not mix during the tuning, because the collateral branch was closed and the balloon remained inflated. This configuration was chosen for the tuning because pigs were assumed to be without collateral flow such that (except for the first seconds of OIS) the vascular bed downstream of the occluding balloon was completely filled with water infused through the OIS catheter.

**Figure 5:**
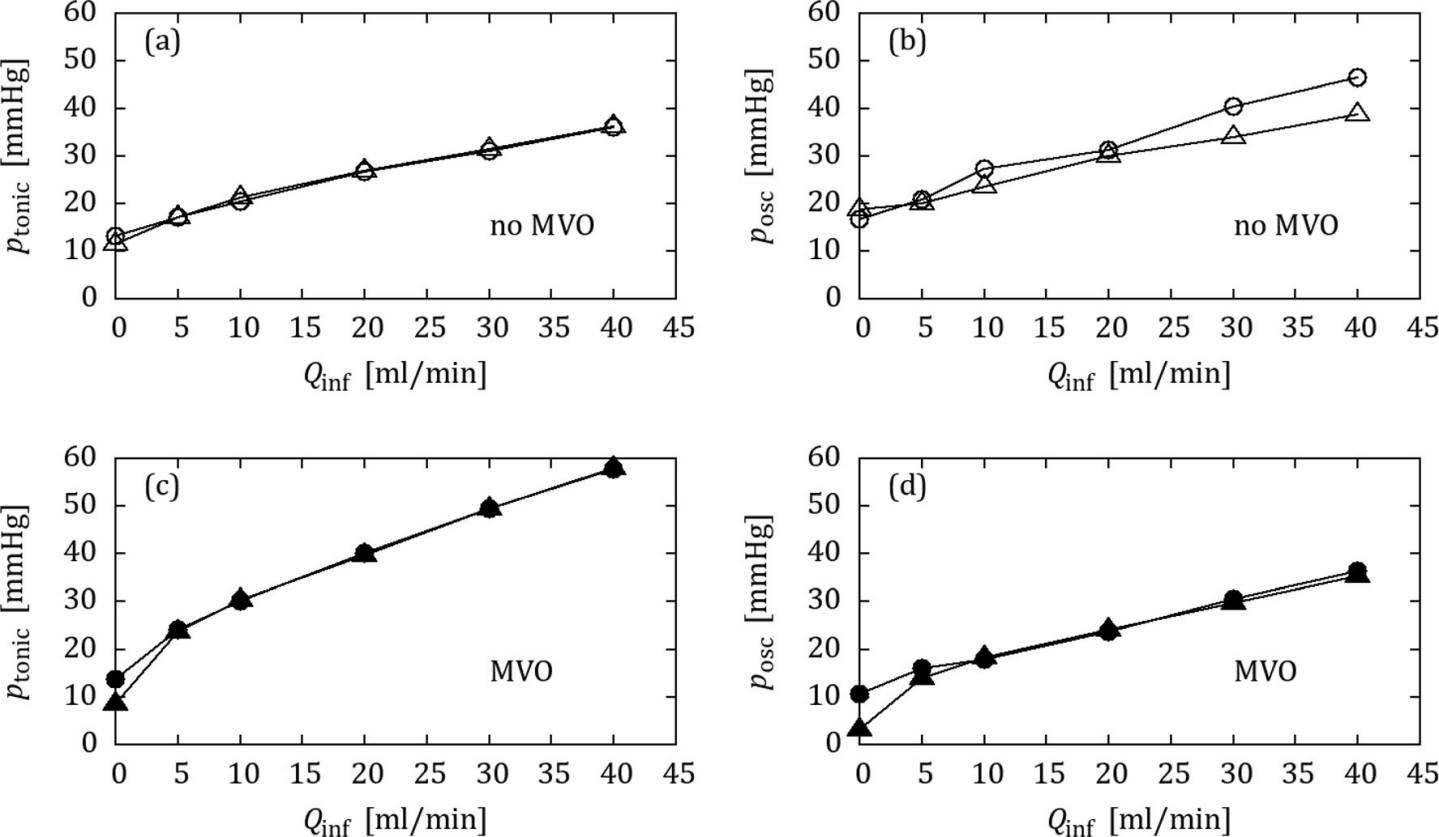
OIS data from the animal trial (△,▲) and from the benchtop model (○,●) matched for the BMF configuration: (a, b) tonic pressure and oscillation amplitude without MVO (open symbols), (c, d) tonic pressure and oscillation amplitude with MVO (filled symbols).

Figure 5 shows the OIS results from the animal and from the benchtop model with BMF after tuning for ${Q}_{\text{coll}}$ = 0 mL/min. It was not possible to carry over the settings from the experiments with water alone, because the presence of BMF in the left-heart mock loop directly affected the dynamics of the IP element. Except for the oscillatory pressure component without MVO (Fig. 5b), the agreement between benchtop model and animal data was again excellent. This confirms the previous finding, that the proposed benchtop model was able to reproduce animal data with and without MVO.

Mixing effects had a transient effect on collateral flow rates. As the infusion flow rate increased with each OIS step, the collateral flow rate dropped initially due to increased distal pressure. However, it recovered to a higher level within the first seconds of each OIS step due to the wash-in of water which lowered the viscosity of the fluid mixture (thereby also lowering the distal pressure). This effect was less pronounced for the higher infusion flow rates ${Q}_{\text{inf}}$ because water dominated the flow configuration and mixing effects were less significant.

**Figure 6:**
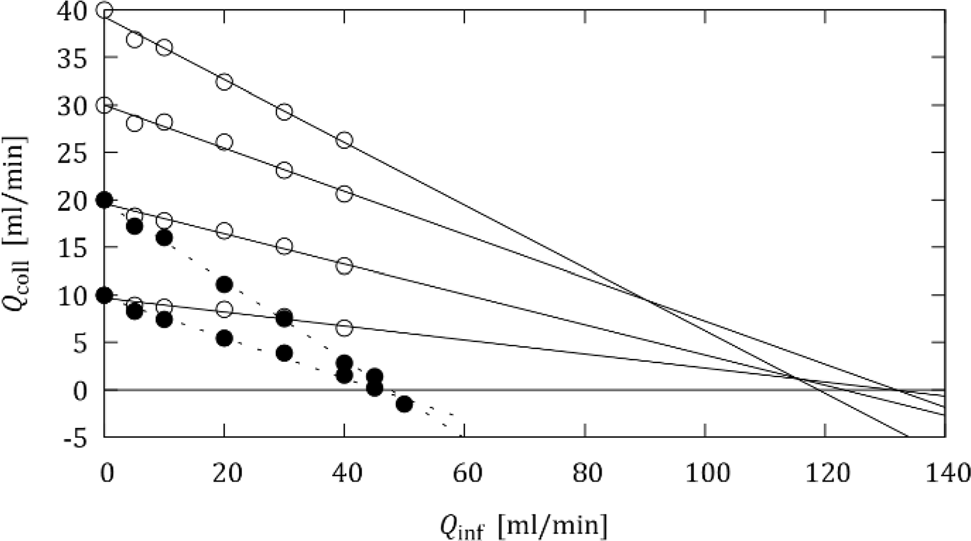
Collateral flow rate ${Q}_{\mathrm{coll}}$ in function of ${Q}_{\text{inf}}$. Experiments in no-MVO configuration (open symbols; solid line) and MVO (filled symbols; dashed lines) for different initial collateral flow rates (${Q}_{\mathrm{coll},0}$= 10, 20, 30, 40 ml/min; for the MVO configuration only ${Q}_{\mathrm{coll},0}$= 10, 20 ml/min could be attained).

Figure 6 shows the mean collateral flow rates for different configurations, where these mean flow rates were computed from intervals excluding the initial transients. These results are very similar to the results for the water experiments (cf. Fig. 3). Linear extrapolation of the results suggests that collateral flow in the healthy configuration without MVO could be suppressed for infusion flow rates of 120 to 140 mL/min. The presence of MVO increased the distal pressure such that collateral flow rates were generally lower. The higher viscosity of BMF increased the distal pressure in the MVO configuration so much, that it was not possible to reach initial collateral flow rates above 20 mL/min. However, it was possible to suppress collateral flow rates with infusion flow rates of approximately 45 mL/min. When the maximum infusion flow rate was increased to 50 mL/min, the collateral flow even reversed, i.e. the resulting distal pressure was higher than the aortic pressure such that there was retrograde flow in the collateral branch.

**Figure 7:**
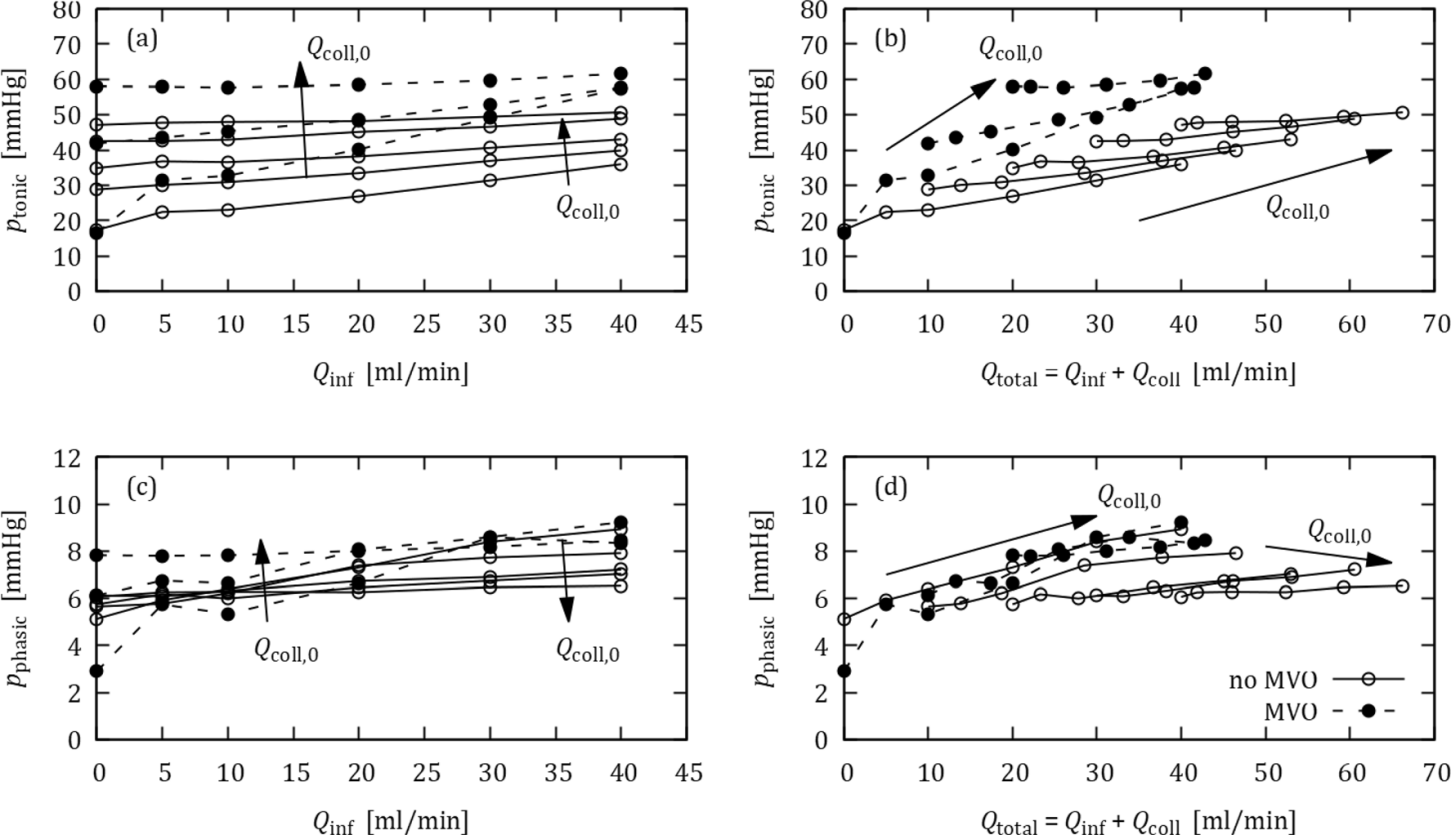
OIS responses with BMF for different initial collateral flow rates ${\mathrm{Q}}_{\mathrm{coll},0}$ (no MVO: 0, 10, 20, 30, 40 mL/min; MVO: 0, 10, 20 mL/min for MVO configuration) with (dashed lines) and without MVO (solid lines): (a) tonic pressure against ${Q}_{\mathrm{inf}}$, (b) tonic pressure against ${Q}_{\mathrm{total}}$ = ${Q}_{\text{inf}}$ + ${Q}_{\mathrm{coll}}$, (c) phasic pressure against ${Q}_{\text{inf}}$, (d) phasic pressure against ${Q}_{\mathrm{total}}$.

Figure 7 summarizes the OIS responses for the experiments with BMF. In general, the results are like the results for the experiments with water (cf. Fig. 4): Higher collateral flow and/or the presence of MVO increases the tonic pressures (Fig. 7a). Replotting the tonic pressure against ${Q}_{\text{total}}$ = ${Q}_{\text{inf}}$ + ${Q}_{\text{coll}}$ (Fig. 7b) separates the MVO results from the no-MVO results, although the curves for different collateral flow rates do not collapse as clearly as for the water experiments (cf. Fig. 4b).

The OIS results for the phasic pressure (Figs. 7c, 7d) clearly exhibited effects of fluid mixing. For the MVO case, the phasic pressure increased with ${Q}_{{\text{coll}},0}$ only for low infusion rates. For the highest infusion rate, ${Q}_{\text{inf}}$ = 40 mL/min, the phasic pressure was almost the same for all ${Q}_{{\text{coll}},0}$, because the effective collateral flow nearly ceased at this infusion flow rate (cf. Fig. 6). Without MVO, higher ${Q}_{{\text{coll}},0}$ even resulted in lower phasic pressures when the infusion flow rate was high. This effect could be attributed to the higher viscosity of the mixed fluid for higher collateral flow rates.

## Discussion

### Model Validation

The effects of collateral flow and fluid mixing on OIS, cannot be easily and systematically studied in an *in vivo* model. We thus developed a coronary circulation model. To validate this model, we first confirmed that it reproduced physiological coronary waveforms with peak flow during diastole,^13^ and next introduced MVO by increasing the distal resistance in the coronary model which reduced the coronary flow rate as expected.

In the second validation step, we identified specific settings for the impedance elements of the coronary model (cf. Fig. 1b) successfully reproducing OIS results from *in vivo* experiments. This was demonstrated for the healthy animal and for the animal with MVO. The OIS results showed tonic and phasic pressure increase with higher infusion rates. If the ratio between tonic pressure and infusion flow rate is interpreted as vascular bed hydraulic resistance, the results indicate that this resistance is flow rate dependent, as it progressively decreases for increasing flow rates. This effect was also visible in the *in vivo* data and is likely due to passive vascular distension at higher perfusion pressures which reduces hydraulic vascular resistance. In the coronary model, this effect was due to the PDR element which introduced a pressure-dependent resistance decreasing with higher flow rate.

When MVO was present tonic pressures were higher. However, the phasic pressure levels for MVO were lower than in the no-MVO setting which agrees with the pig data.

The validation experiments were carried out with and without BMF. The agreement between results from the *in vivo* experiments and from the benchtop model were very good for both configurations (cf. Figs. 2, 5), such that the proposed benchtop experiment was found to be a good model of coronary flow with and without MVO.

### Collateral Flow

To assess the effect of collateral flow, water experiments were repeated for different collateral flow rates while the settings for the other impedance elements in the coronary model were held constant. Increased collateral flow led to increased tonic pressure (cf. Fig. 4). In the OIS, this effect could mask the MVO effect which is also marked by increased distal pressure. In practical clinical settings, it is difficult to decide whether an OIS response showed increased pressures due to MVO or due to collateral flow. This is a problem in patient management because MVO and collateral flow can have opposite effects on patient outcome after myocardial infarction. Figure 4b suggests a remedy for this problem. It shows that the effect of collateral flow can be eliminated from the analysis if the tonic pressures are plotted against the total coronary flow rate, ${Q}_{\text{total}}$ = ${Q}_{\text{inf}}$ + ${Q}_{\text{coll}}$. However, this remedy is of little practical relevance as collateral flow rates are typically unknown in patients.

**Figure 8:**
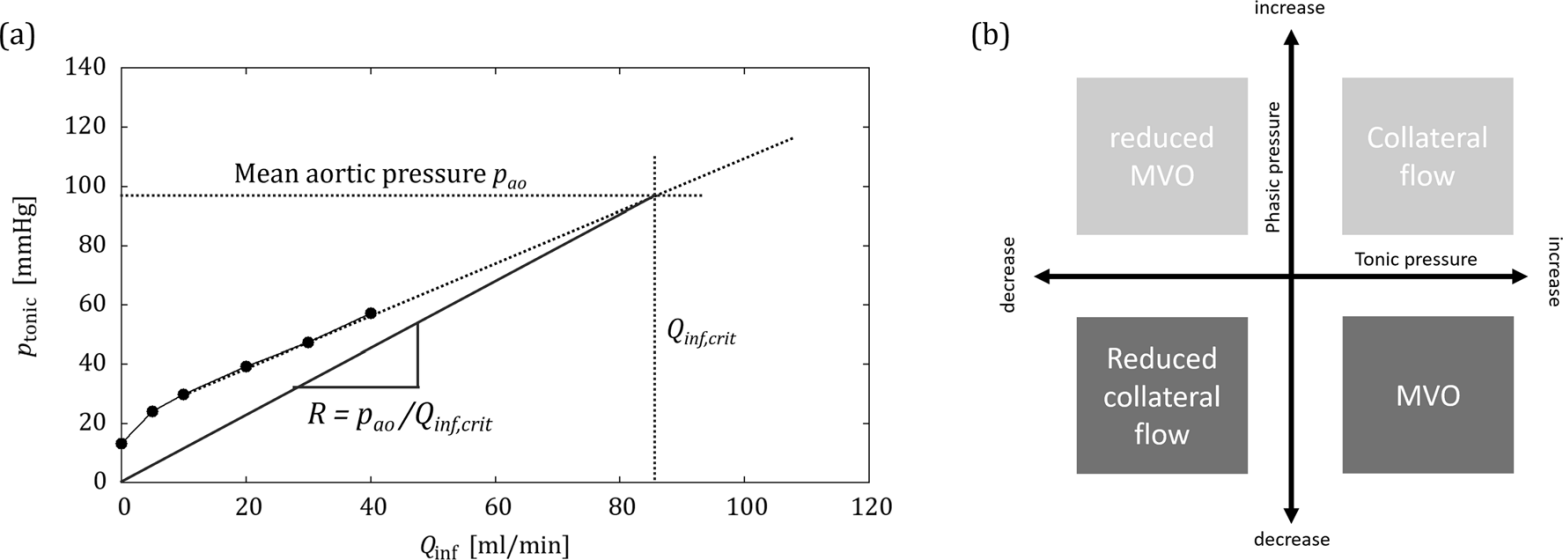
Concepts for mitigating the effect of collateral flow on the OIS: (a) estimation of ${Q}_{\mathrm{inf},\mathrm{crit}}$ by extrapolation of tonic pressures, (b) effect of collateral flow and MVO on tonic and phasic pressure increase/decrease.

Another possible strategy to mitigate the collateral flow effects comes from the observation that collateral flow decreased with increasing infusion flow rate. It even stopped when the infusion flow rate was sufficiently increased (cf. Fig. 6). This point corresponded to a configuration where the tonic pressure had reached the level of the mean aortic pressure ${p}_{ao}$, such that the pressure difference across the collateral branch was zero and there was no collateral flow. The total flow in the distal vascular bed was then equal to the infusion flow rate. Therefore, the ratio of the tonic pressure to that critical infusion flow rate ${Q}_{\text{inf,crit}}$ corresponds to the actual hydraulic resistance of the distal vascular bed. Unfortunately, this critical infusion flow rate ${Q}_{\text{inf,crit}}$, at which collateral flow stops, is not known a priori for an actual patient. Moreover, this flow rate will typically be beyond the maximum infusion flow rate of 40 ml/min which was chosen for patient safety reasons. Nevertheless, it may be possible to estimate ${Q}_{\text{inf,crit}}$ by extrapolating the curves for the tonic pressure measured with the OIS to the level of the aortic pressure ${p}_{ao}$ (Fig. 8a). This point on the extrapolated curve yields an estimate for ${Q}_{\text{inf,crit}}$. The vascular resistance could then be computed as ${R={p}_{\text{ao}}/Q}_{\text{inf,crit}}$.

A second approach to mitigate the effect of collateral flow on the OIS, is based on the opposing effects of MVO and collateral flow on phasic pressure. Figure 4c shows that MVO decreased phasic pressure, while collateral flow increased it. Therefore, an increase in tonic pressure should be associated with MVO only if there is also a decrease in phasic pressure. If both pressure components increase, the effect is due to collateral flow (Fig. 8b). However, these effects for the phasic pressure are much less clear in the experiments with BMF and may even be reversed for higher infusion flow rates (Fig. 7c).

The applicability of these two approaches for distinguishing between effects of collateral flow and of MVO will be assessed in future studies which could also include other clinical parameters such as the collateral flow index (CFI).^11,19^

### Fluid Mixing

The infusion of a water-like fluid with lower viscosity through the catheter into the coronary flow model filled with BMF had a direct effect on the distal pressures measured during the OIS. In configurations without collateral flow, the effect was limited in time as the BMF in the distal branch was eventually replaced with water. The situation was more complex with collateral flow because of continued inflow of BMF through the collateral branch which mixed with the infused water. This changed the apparent viscosity in the distal branch and thereby affected the OIS pressure measurements. In the present experiments, it was found that a steady state with constant mixing ratio was reached after 10-15s. However, this steady state had to be re-established for every infusion flow rate. Therefore, it is important to have sufficiently long intervals of constant ${Q}_{\text{inf}}$ in the OIS to reach a steady state from which the tonic and phasic pressure levels can be estimated.

Apart from the transient mixing effects described above, the change of infusion flow rate and collateral flow rate during the OIS (cf. Fig. 6) led to different steady-state mixtures of BMF and water for each OIS step. These mixtures had higher viscosity than water which led to higher hydraulic resistance in the coronary model and to higher distal pressures during OIS. This affected the OIS results such that the replotting of the pressure against the total flow rates ${Q}_{\text{total}}$ (Figs. 7b and 7d) did not result in a full separation of MVO from no-MVO data as it could be observed for the experiments with water only (Figs. 4b and 4d). This effect was strongest for low infusion flow rates ${Q}_{\text{inf}}$. For higher ${Q}_{\text{inf}}$ (and, in consequence, for lower ${Q}_{\text{coll}}$) the infusion of water dominated the mixture such that there was less variation in viscosity as ${Q}_{\text{inf}}$ further increased. Therefore, the collapse of the curves improved with higher ${Q}_{\text{inf}}$.

Despite these additional complications introduced by fluid mixing, the basic observations from the water-only experiments remained valid.

### Limitations

The coronary vascular system is modelled by impedance elements lined up along a single tube (lumped-element model), which implies the assumption that mechanical properties of multiple vessels can be lumped into a single functional element. This is certainly a simplification, and the model may not be able to reproduce complex nonlinear phenomena of the coronary system. However, the successful validation of the coronary model using OIS data from animals suggests that the complexity of the proposed lumped-element model is sufficient to mimic the coronary hemodynamics.

The circulation model did not include any coronary autoregulation mechanisms which can modify vascular resistance by vasoconstriction or vasodilation. This limitation of the model may be benign for this study because the balloon occlusion and the infusion of isotonic buffered solution during OIS induces hyperemia and full vasodilation in the animal, such that autoregulatory mechanisms are negated during OIS. Note that this may not be the case anymore in humans where collateral flow could reduce hyperemic effects during OIS.

The first systolic peak of the coronary waveform in the no-MVO configuration (Fig. 1d) is higher than reported in literature.^13^ The first systolic peak is attributable to the strong increase in aortic pressure at early systole which drives the flow in the proximal coronary model. This pressure increase is partially negated by the simultaneous increase in hydraulic resistance due to the IP element in the distal part of the model which models the compression of the intramyocardial vessels. Nevertheless, the compliance of the proximal tubing in the coronary phantom allows for a first systolic peak. This peak may be too high in this model because of the length of the tubing used for the LAD model which provides more compliance volume than a real pig LAD. The long LAD tubing in the model also leads to a higher priming volume which could explain why the transient for fluid mixing is longer than in the animal experiments.

## Concluding Remarks

A multi-scale benchtop model for coronary circulation model was described, validated and used to study a catheter-based diagnostic method for cardiac MVO which uses an occlusion–infusion sequence (OIS) to estimate the resistance of the vascular bed. The assessment of this method is difficult using *in vivo* models alone, because of the limited ability to systematically define and control model parameters (e.g., heart rate, blood pressure) and due to inter-individual variations in physiology and anatomy. The presented *in vitro* benchtop model can overcome these limitations because it provides a controlled, reproducible, tunable and calibrated environment to assess the hemodynamics of the coronary circulation in real-time using systematic parameter studies.

We showed that collateral flow in the coronary circulation can lead to misleading results from the OIS, because collateral flow resulted in increased distal pressures which could be misinterpreted as increased hydraulic resistance due to MVO. Further, this study investigated effects of fluid mixing due to the simultaneous presence of blood and water-like fluid (infused through the OIS catheter) in the coronary circulation. Results indicated transient mixing processes during the OIS which may lead to incorrect pressure measurements, unless the OIS steps provide enough time for the system to reach a steady state with a constant mixing ratio.

The study highlights that not only the tonic pressure should be considered, but that also the phasic component of the pressure signal may hold relevant information on the hemodynamics of the coronary circulation. Finally, two concepts were proposed to better differentiate between MVO and collateral flow when interpreting OIS results. This study may be a used as basis to identify clinical diagnostic markers for MVO severity using the catheter-based OIS approach.

## Supplementary Information

Below is the link to the electronic supplementary material.

Supplementary file1 (PDF 430 kb)
