# Supplementary material for: Effect of Collateral Flow on Catheter-Based Assessment of Cardiac Microvascular Obstruction
Source: Ann Biomed Eng. 2022 May 31;50(9):1090–102. doi: 10.1007/s10439-022-02985-2 (PMC9363345; doi:10.1007/s10439-022-02985-2)
Supplement: Supplementary file 1 — Supplementary file1 (PDF 430 kb) [file 10439_2022_2985_MOESM1_ESM.pdf]

Appendix of *Thirugnanasambandam et al., Annals of Biomedical Engineering, 2022, doi: 10.1007/s10439-022-02985-2*

Tuning of the benchtop model

The benchtop model was tuned by adjusting the impedance elements shown in Figure 1b and Figure S1. The procedure consisted of five sequential steps (Figure S2) to match the flow-pressure response of an animal model:

1. The resistance element and the compliance element in the left-heart mock loop are adjusted to match the aortic pressure and heart rate to that of the animal.
2. The tonic pressure (p_{tonic}) from the benchtop model is matched to that of the animal model by simultaneously adjusting the following two elements:
    a. The external pressure p_{ext} of the PDR element is adjusted until p_{tonic} corresponding to Q_{inf} = 20 and 30 ml/min match those of the animal model.
    b. The resistance of the static resistor element is adjusted until p_{tonic} corresponding to the remaining infusion flow rates (Q_{inf} $=$ 5, 10, 40 ml/min) matches those of the animal model.
3. The oscillation amplitude (p_{osc}) from the benchtop model is tuned to animal data using a combination of the following steps:
    a. The proximal compliance is adjusted until the minimum distal pressure p_{min} = min$\{p_d\}$ matches that of animal data for each flow rate.
    b. The distal compliance element is adjusted to match the oscillation amplitude of the pressure waveform by tuning the maximum pressure p_{max} = max$\{p_d\}$.
4. The outlet height is adjusted to selectively tune p_{tonic} at lower infusion flow rates (Q_{inf} $=$0 ml/min, 5 ml/min) and the oscillation amplitude p_{osc}.

These steps may have to be repeated iteratively to achieve a good match of the flow-pressure curve to the animal data.

This tuning procedure was first used to tune the model to a healthy control. To model the effect of MVO only the PDR element (to offset the curve), static resistor element (to increase the slope) and the outlet height (to adjust the oscillation amplitude) were adjusted in the benchtop model, whereas the other impedance elements were left unchanged.

The following settings were used for the experiments reported in this paper: compliance air volume 2ml (proximal compliance element), 1ml (distal compliance element); p_{ext} $=$ 8kPa (80cm water column); outlet height = 9cm.

Supplementary Figures of ***Thirugnanasambandam et al., Annals of Biomedical Engineering, 2022, doi: 10.1007/s10439-022-02985-2***

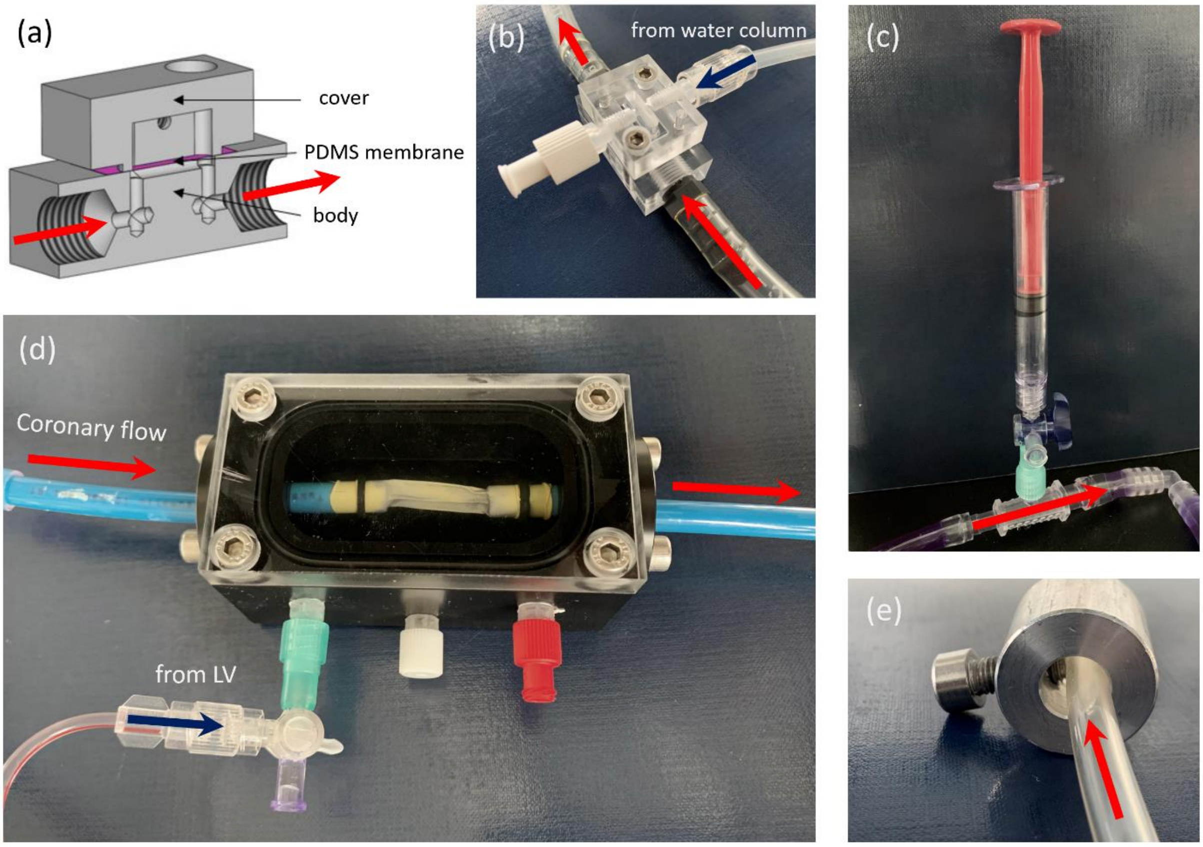

Figure S1: (a) Sectional drawing of pressure-dependent resistor (PDR) (coronary flow through body element; separated by PDMS membrane from cover element where pressure p_{ext} is imposed). (b) PDR element connected to coronary tubing and to water column for imposing p_{ext}. (c) Syringe used as compliance element. (d) Intramyocardial Pump (IP) connected to coronary tubing and to LV for imposing p_{LV}. (e) Adjustable resistance element. (Red arrows: coronary flow).

**Element**	**Effect of tuning**
(1) LH flow loop	Matches heart rate and aortic pressure
(2a) PDR element	Shifts OIS flow-pressure response curve vertically without changing slope
(2b) Static resistor	Changes the slope of the OIS flow-pressure response curve
(3) Compliance elements	↑ Proximal compliance → ↓ p_{tonic}, ↓ p_{max}, ↑ p_{min}, ↓ p_{phasic}
	↑ Distal compliance → ↓ p_{tonic}, ↓ p_{max}, ≈ p_{min}, ↓ p_{phasic}
(4) Outlet height	↑ Outlet height → ↓ p_{tonic}, ↑ p_{phasic} (at lower Q_{inf}); ≈ p_{tonic} (at higher Q_{inf})

Figure S2: Tuning procedure for the impedance elements of the coronary flow model.
